# Root biomass and root morphological traits of three shrub species: Implications for the soil anti-scouring resistance of the ecological slope

**DOI:** 10.1371/journal.pone.0288848

**Published:** 2023-11-16

**Authors:** Mingxin Zhou, Guoyong Yan, Yibo Li, Di Chen, Chao Yan, Nan Wang, Chao Jia, Qinggui Wang, Yajuan Xing

**Affiliations:** 1 Heilongjiang Academy of Forestry, Harbin, China; 2 Northeast Forestry University, Harbin, China; 3 Heilongjiang Institute of Construction Technology, Harbin, China; 4 School of Life Sciences, Qufu Normal University, Qufu, China; 5 Heilongjiang Polytechnic, Harbin, China; Qinghai University, CHINA

## Abstract

The purpose of this study was to determine which shrub species will enhance soil anti-scouring resistance on an ecological slope. Root traits and soil anti-scouring resistance of three shrubs (Amorpha fruticosa Linn (AFL), Swida alba Opiz (SAO) and Lespedeza bicolor Turcz (LBT)) were measured. Results showed that root biomass and root morphological traits of three shrubs were significantly correlated with the soil anti-scouring resistance index. According to the composition characteristic values, root morphological traits among the three shrubs had a high contribution rate. Under two slopes and two rainfall conditions, when root biomass and root morphological traits (e.g., root length, root volume and root surface area) were identical, AFL had the highest soil anti-scouring resistance index. These results suggested that root biomass and morphological traits of AFL had more significant effects on soil anti-scouring resistance comparing with SAO and LBT. Therefore, in engineering practice, AFL with stronger soil anti-scouring resistance can be selected as slope plants.

## 1. Introduction

Rainfall is one of the main reasons for the instability of expressway ecological slope [1]. This instability is caused by the erosion power of the rain [2]. For expressway slopes, shallow landslide caused by rainfall is one of the most common geological disasters [3]. Practice shows that, expressway slopes under conditions of continuous rainfall, surface soil is subject to erosion by runoff and rainfall. The instability of expressway ecological slope has adverse effects on driving safety [4].

Previous studies found that vegetation cover was very important for controlling erosion [5–7]. Ecological slope protection technology is used to improve slope stability and prevent shallow landslide [8]. People pay more attention to ecological slope protection technology, aiming to stabilize the shallow slope, reduce the risk of shallow landslide caused by rainfall, and improve the ecological environment [9, 10]. The reasons of expressway ecological slope instability caused by rainfall are complicated [11].

Previous studies found that root biomass and root morphological characteristics played an important role in the enhancement effect of soil anti-scouring resistance [12]. The effect of the root system in enhancing soil anti-scouring resistance was weakened with increasing rainfall intensity [13]. Vegetation plays a very important role in soil erosion control and is the most effective and fundamental method in soil and water conservation [14]. The enhancement of soil anti-scouring resistance mainly depends on the winding, consolidation and serial soil action of roots, this effect makes the soil have a high-water stability structure and impulse resistance strength, which is not easy to be taken away by runoff [15]. Some studies have been conducted on the role of plant roots on soil retention, but traditional studies have focused on the effects of aboveground biomass, but less attention is paid to the role of underground biomass [16]. More studies are needed to verify the effects of root morphological characteristics on soil anti-scouring resistance.

Root anti-scouring resistance is a macroscopic manifestation of shrub roots in its mechanical properties. However, the root morphological characteristics are the intrinsic nature of the root generation of a macroscopic phenomenon. Studying root morphological characteristics can help to explain the mechanical properties from the perspective of shrub roots, and reveal the mechanism.

We set out to fill in the knowledge gaps by determining the following: the dominant shrubs can be beneficial to the slope protection by comparing the soil anti-scouring resistance index under two rainfalls conditions with slopes of both 30° and 60°. In addition, by studying the relationship between root morphological characteristics and mechanical indicators, revealed the internal mechanism affecting soil anti-scouring resistance. Finally, through the principal component analysis, we determined the root morphological characteristics affecting the mechanical indicators.

In this study, three typical types of shrubs in the northeast were selected by field investigation. The three shrub species are *Amorpha fruticosa* Linn. (AFL); *Lespedeza bicolor* Turcz (LBT); *Swida alba* Opiz (SAO). We assumed that mechanical reinforcement of root traits in different response directions would differ based on species traits. The results of this study provide planting plant options for preventing rain erosion on road slopes.

## 2. Materials and methods

### 2.1. Study sites expressway introduction

Hatong Expressway starts in Harbin and ends in Tongjiang. The traffic mileage is 595 km. The Hatong Expressway was built in 1997. This expressway was extended in 2005, and its slopes were planted with shrubs to increase slope stability. This expressway is located on the south of the Songhua River, and on the northwest of the Zhangguangcai Ridge that the branch of Changbai Mountain. The line position area is mostly mountainous and hilly. The belt along the expressway is mostly hilly, undulating terrain. On the Hatong Expressway, which connects Harbin to Jiamusi in northeastern China, a site test was conducted. In this study area, the winters are longer, the summers are shorter, the winters are cold, and the summers are warm. This is typical of a temperate continental monsoon climate. The average annual rainfall at the study site is about 300 mm to 700 mm. Rainfall is more concentrated, the rainfall time is generally concentrated in July-September, accounting for more than 60% -70% of the annual rainfall. There are a number of challenges associated with this type of rainfall in the study area. The study area map is shown in S1 Fig in S1 File.

### 2.2. Experimental design and sampling

At the aspect of selection with tree species, the research followed the principles of common, suitable and engineering precedent. After field investigation and reference, the species of study to be determined were: Amorpha fruticosa Linn (AFL), Swida alba Opiz (SAO) and Lespedeza bicolor Turcz (LBT). In this experiment, we collected shrubs from the same slope of site conditions. The main shrub characteristics of the study plots are as follows (Table 1).

**Table 1:** Characteristics of important shrub species in the study area.

Species	Mean height/cm	Mean coverage/%	canopy/cm	basal diameter/cm	Root biomass g/plant
AFL	178.28±3.76	96.5±1.02	135.67±9.66	1.87±0.12	40.07±0.34
LBT	221.15±5.66	92.4±0.99	153.66±11.23	2.25±0.07	42.98±0.84
SAO	211.45±2.66	92.7±1.42	166.29±10.57	1.92±0.09	49.71±0.56

In early June 2020, we selected three years shrub which were cutted by the slope of Harbin-Jiamusi section, and potted them. The containers were selected with 50 cm × 30 cm plastic flowerpots. The soil used for planting was the base soil of the cutting slope of Harbin-Jiamusi section of Hatong Expressway. The potted shrubs were 50 flowerpots for each type, and planted one shrub in each flowerpot. During the cultivation period, we performed routine management. In early September 2021, we performed the soil anti-scouring resistance test. After the soil anti-scouring resistance test, plants were removed from the flowerpots to mensurate shrub root biomass and morphological traits. The final conclusion was drawn using the experimental data and statistical analysis. S2 Fig in S1 File shows the flowchart of the methodology.

#### 2.2.1. Soil anti-scouring resistance index

Soil anti-scouring resistance refers to the mechanical damage and traffic capacity of the soil to the water flow, which is mainly related to the soil physical properties and external biological factors [17]. The experiments performed in this study were modified from the original soil scour pool (S3 and S4 Figs in S1 File). In order not to destroy the soil structure, we made a 20cm × 18cm × 10cm sampler, the slope of the resistant tank was designed to be 30° and 60°. According to the average value of the potential runoff velocity (2mm/min) caused by the typical moderate storm in the study area, the simulated runoff flux was designed. Adjust the outlet flow rate and set the outlet flow rate to 2L/min and 0.5L/min respectively. The outlet flow rate was set at 2L/min and 0.5L/min to simulate the precipitation of heavy rain and light rain in the study area. During the scouring process, the maximum scouring duration was 10min. The scouring duration was set as 10min, which was the average rainstorm time in the study area. If the scouring was completed within 10 minutes, recorded the time when the scouring was completed. Before the experiment, we cut the ground part of the shrub and cut the flowerpots. We performed three replicate experiments for each condition. This paper used the water quantity Q required to wash away 1 g of soil with a certain water flow rate to indicate the soil anti-scouring resistance. The more water required for scouring 1g soil, the stronger the anti-scouring ability of the root-soil complex. On the contrary, if the less water required for scouring 1g soil, the weaker the anti-scouring ability of the root-soil complex. Therefore, the amount of water required to scour 1g soil with a certain water flow can be used as an important indicator to measure the anti-scouring ability of the root-soil complex. The specific operation steps of soil anti-scouring resistance index determination were as follows (S4 Fig in S1 File): (1) Before the scouring test, we putted the potted plants on the water surface and let the soil absorb water to saturation; (2) After taking out the potted plants, first stand and remove the gravity water until no obvious water drops flow out to obtain the same soil moisture content; (3) Cut the plastic flowerpot, cut off the aboveground part of the plant, we used the soil sampler to take out the soil block with complete plant roots, and put the soil block with complete plant roots together with the soil sampler into the washing tank for washing; (4) then, we collected and recorded the amount of soil scoured in unit time, dry at 105°C, and weigh, and recorded it as ΔW (mass of soil washed away during scouring). The formula was as follows: C_s_ = Q/ ΔW. In formula: C_s_- Soil anti-scouring resistance index (L/g); Q- Water quantity used for scouring (L) (Constant outlet flow rate); ΔW- Soil quality which washed away (g).

#### 2.2.2. Root biomass and root morphological traits

After the soil anti-scouring resistance test, we measured the root biomass (RB). The plants were removed from the flowerpots, and washed the roots with deionized water. Placed to dry in the laboratory, and separated the above-ground and the under-ground parts. Placed the under-ground parts into a brown paper envelope, put it in the oven with a constant temperature of 80°C, and dried to constant quality. We measured the biomass of the underground part at this time. According to the indoor variable-head permeability test method, the permeability test was carried out on the soil samples containing roots under various root biomass in the mixed rooting state. The water content in the test was set at 25%, the root diameter was 0.5–1.5mm, and the void ratio was 0.99. The permeability coefficient of the soil containing roots under different root biomass was obtained.

And then, we measured the root morphological traits. The residual soil particles of the roots were carefully washed in deionized water with forceps in the laboratory. We selected live roots by identifying the color, elasticity, structure, and texture of the test shrub roots. Root samples for morphological analysis were carefully dissected with forceps by branch order, following the root architecture work of Fitter [18]. The roots of each shrub were scanned by a scanner (Expression 11000XL) with a resolution of 600 dpi. We measured the total root surface area (TRSA), total root volume (TRV), and total root length (TRL) by WinRHIZO-TronMF 2012 software (root system analysis software).

## 3. Statistical analyses

Regression analysis was used to investigate the relationship between soil anti-scouring resistance index(C_s_) and root biomass (RB), total root surface area(TRSA), total root volume(TRV), and total root length(TRL) at different slopes. The above data analysis was done through the ggplot2 package of the R. The difference in soil anti-scouring resistance index between different shrub species was investigated by using the Tukey-Kramer-HSD one-way analysis of variance (ANOVA) method. The effects of the interaction between different plants, slopes, and different parameters were evaluated by multiway ANOVA. Principal component analysis was used to analyze the main factors affecting soil anti-scouring resistance index. All data analysis was performed using the SPSS software version22.0. All figures were conducted using SigmaPlot 12.5.

## 4. Results

### 4.1. Soil anti-scouring resistance index

**Fig 1:**
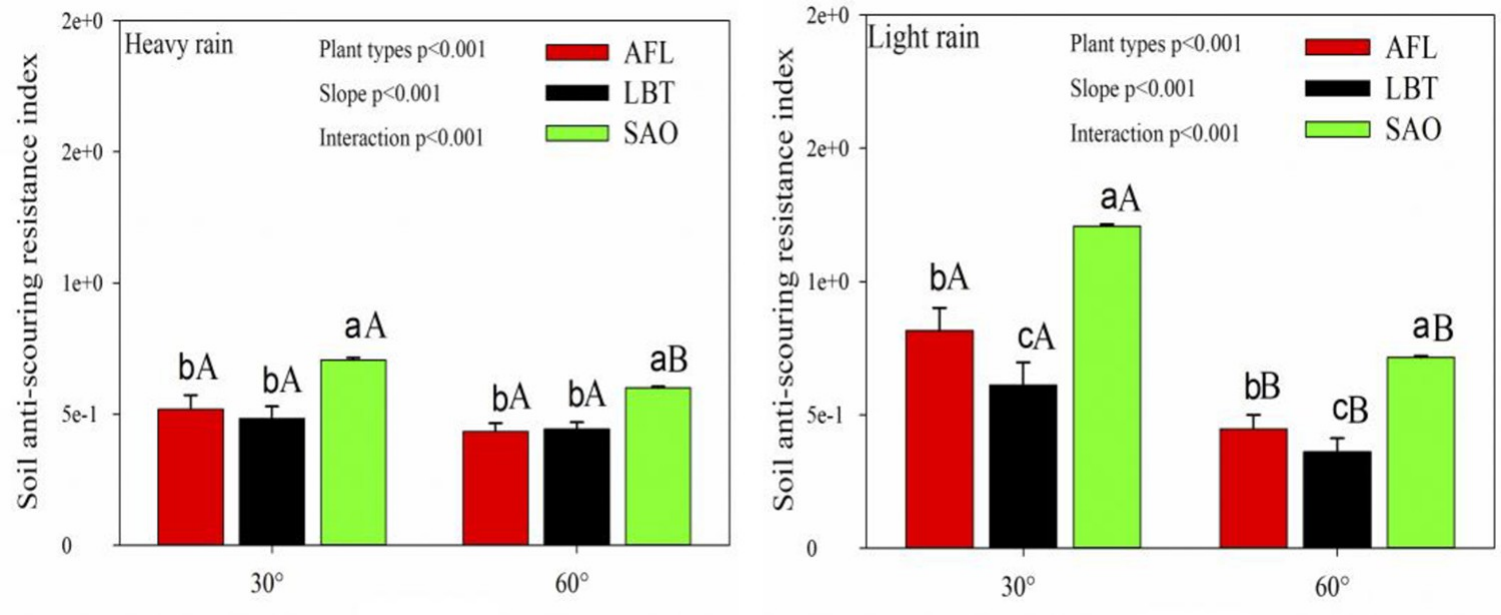
Soil anti-scouring resistance index at different slopes and different rainfall among three plant types (mean ± SE). Different lowercase letters represent statistical significances in different plant types for same soil slope; different capital letters represent statistical significances in same plant type for different soil slopes. AFL, *Amorpha fruticosa* Linn.; LBT, *Lespedeza bicolor* Turcz.; SAO, *Swida alba* Opiz.

The roots of all 3 shrubs showed strong soil anti-scouring resistance under two rainfalls conditions with slopes of both 30° and 60° (Fig 4). Under heavy rain, the soil anti-scouring resistance index of SAO was significantly higher than that of AFL and LBT with slopes of both 30° and 60° (Fig 1). Under light rain, the soil anti-scouring resistance index of SAO was significantly higher than that of AFL and LBT, and AFL was significantly higher than LBT with slopes of both 30° and 60° (Fig 1). In addition, for the same shrub, with increasing slope, the soil anti-scouring resistance index decreased. There was a significant negative correlation between soil anti-scouring resistance index and slope (Fig 1). Moreover, plant type, slope, and their interaction had significant effects on all soil anti-scouring resistance index for all slopes (Fig 1).

### 4.2. Effects of root properties on soil anti-scouring resistance index

**Fig 2:**
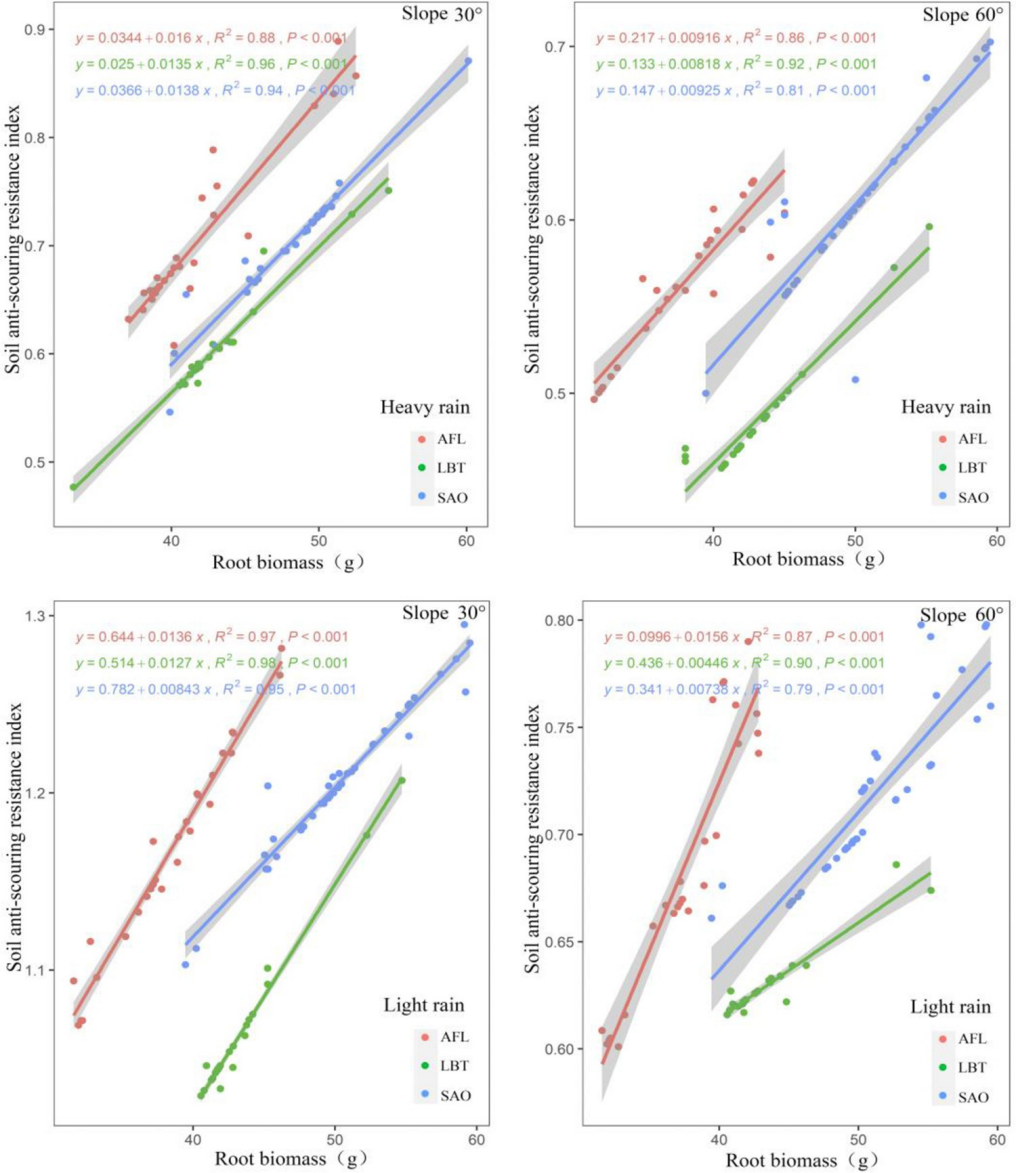
Under heavy and light rain, relationship between soil anti-scouring resistance index and root biomass at different slopes for three shrub species.

There was a positive relationship between soil anti-scouring resistance index and root biomass in all three shrubs under two rainfalls conditions with slopes of both 30° and 60° (Fig 2). Under heavy and light rain, when the shrub root biomass was the same, AFL had the highest soil anti-scouring resistance index, followed by SAO, and LBT with slopes of both 30° and 60° (Fig 2). Moreover, plant type, root biomass, and their interaction had significant effects on all soil anti-scouring resistance index for all slopes (Fig 2). When the root biomass in the soil was less than 25%, the permeability coefficient of the soil with roots increased with the increase of the root biomass, showing a positive correlation (S1 Table in S1 File). The permeability coefficient of soil with roots reached a peak when the root biomass reached 25%. When the root biomass in the soil was higher than 25%, the permeability coefficient of the soil with roots decreased with the increase of the root biomass, showing a negative correlation.

**Fig 3:**
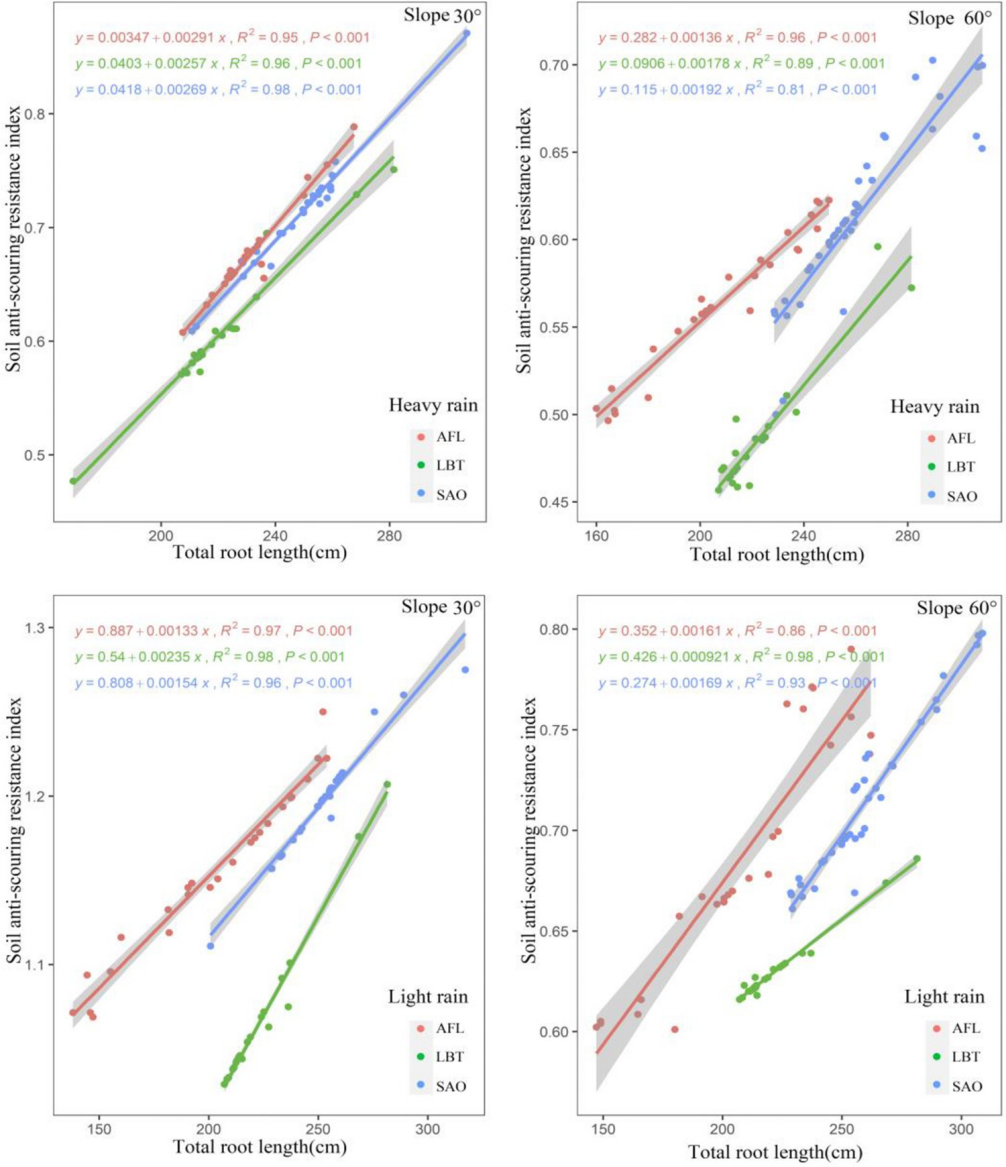
Under heavy and light rain, relationship between soil anti-scouring resistance index and total root length at different slopes for three shrub species.

There was a positive relationship between soil anti-scouring resistance index and total root length in all three shrubs under two rainfalls conditions with slopes of both 30° and 60° (Fig 3). Under heavy and light rain, when the shrub total root length was the same, AFL had the highest soil anti-scouring resistance index, followed by SAO, and LBT with slopes of both 30° and 60° (Fig 3). Moreover, plant type, total root length, and their interaction had significant effects on all soil anti-scouring resistance index for all slopes (Fig 3).

**Fig 4:**
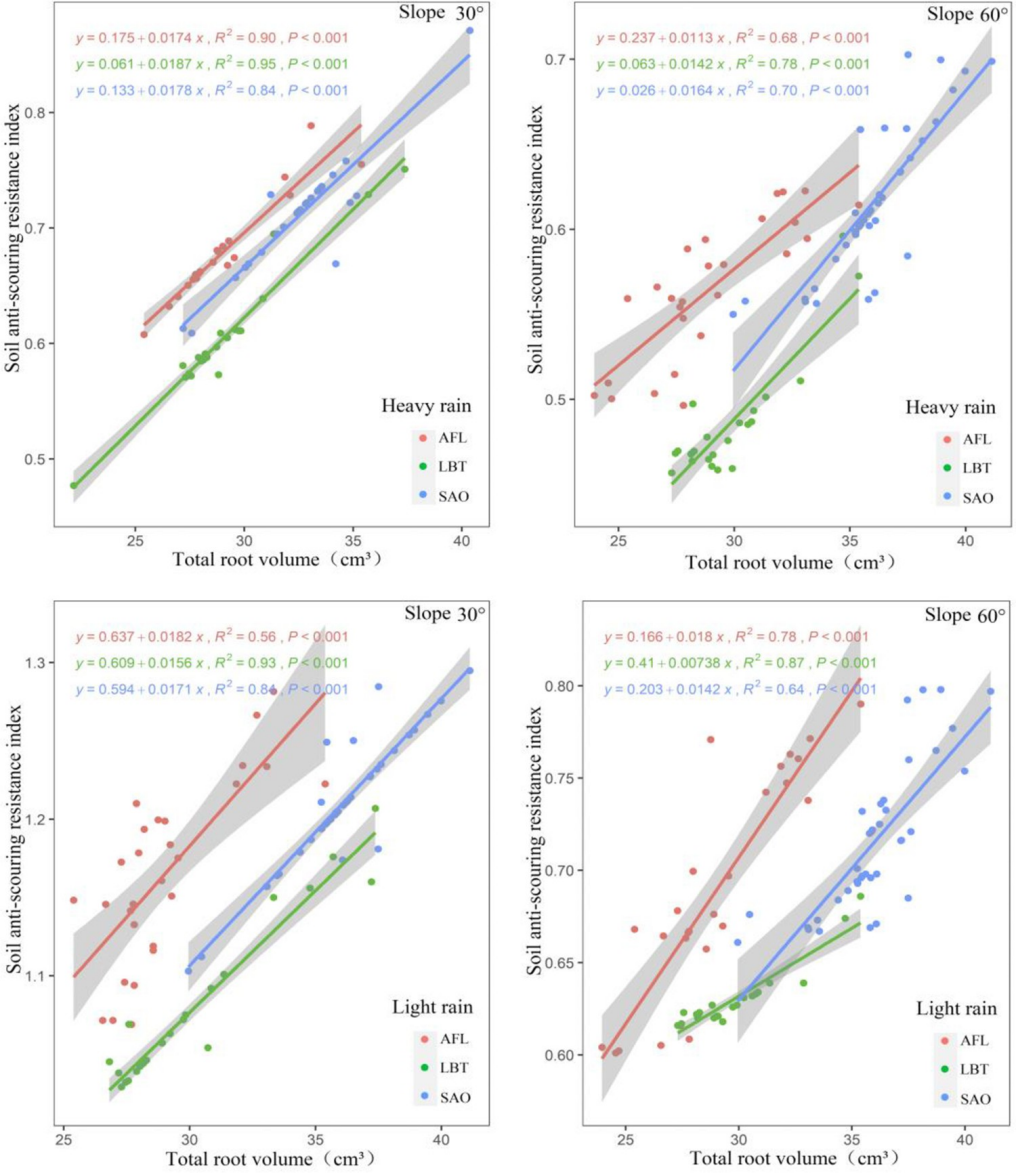
Under heavy and light rain, relationship between soil anti-scouring resistance index and total root volume at different slopes for three shrub species.

There was a positive relationship between soil anti-scouring resistance index and total root volume in all three shrubs under two rainfalls conditions with slopes of both 30° and 60° (Fig 4). Under heavy and light rain, when the shrub total root volume was the same, AFL had the highest soil anti-scouring resistance index, followed by SAO, and LBT with slopes of both 30° and 60° (Fig 4). Moreover, plant type, total root volume, and their interaction had significant effects on all soil anti-scouring resistance index for all slopes (Fig 4).

**Fig 5:**
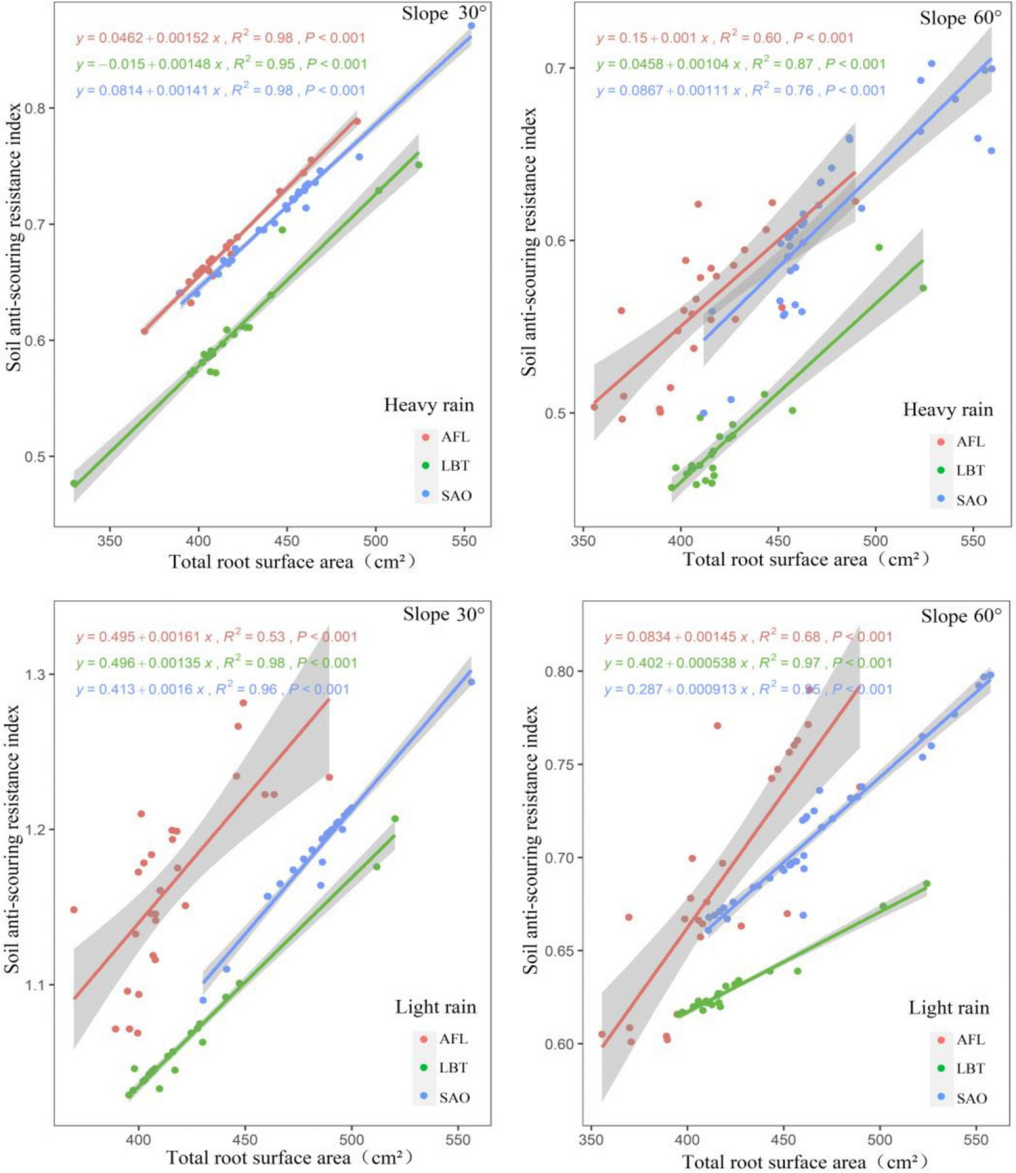
Under heavy and light rain, relationship between soil anti-scouring resistance index and total root surface area at different slopes for three shrub species.

There was a positive relationship between soil anti-scouring resistance index and total root surface area in all three shrubs under two rainfalls conditions with slopes of both 30° and 60° (Fig 5). Under heavy and light rain, when the shrub total root surface area was the same, AFL had the highest soil anti-scouring resistance index, followed by SAO, and LBT with slopes of both 30° and 60° (Fig 5). Moreover, plant type, total root surface area, and their interaction had significant effects on all soil anti-scouring resistance index for all slopes (Fig 5).

### 4.3. Analysis of the main factors affecting the soil anti-scouring resistance index

To study the effect of shrub roots on soil anti-scouring resistance, PCA was used to select for key factors, which were ranked by the size of the factor characteristic value. The first principal component had a high factor load with the TRL, TRV, and TRSA, which was called the root morphology factor. The second principal component had a higher factor load with the root biomass, called the biomass factor (Tables 2–5). According to the component characteristic value and contribution rate, the shrub root morphological factor explained 91.238% (Table 2), 94.078% (Table 3), 91.725% (Table 4), and 76.742% (Table 5). Under heavy and light rain, root morphological factors played a relatively greater role on soil anti-scouring resistance characteristics with slopes of both 30° and 60°.

**Table 2:** Principal component rotation factor load and public factor variance of shrub root morphological traits at 30 slope under light rain.

Research indicators	Principal component		communality
	1	2	
Root biomass	0.434	0.898	0.994
Total root length	0.851	0.517	0.992
Total root volume	0.893	0.447	0.997
Total root surface area	0.868	0.494	0.997
Characteristic value	3.650	0.332	
Variance %	91.238	8.294	
Cumulative variance%	91.238	99.532	

**Table 3:** Principal component rotation factor load and public factor variance of shrub root morphological traits at 60 slope under light rain.

Research indicators	Principal component		communality
	1	2	
Root biomass	0.549	0.828	0.997
Total root length	0.801	0.589	0.985
Total root volume	0.888	0.442	0.983
Total root surface area	0.871	0.490	0.998
Characteristic value	3.763	0.193	
Variance %	94.078	4.827	
Cumulative variance%	94.078	98.906	

**Table 4:** Principal component rotation factor load and public factor variance of shrub root morphological traits at 30 slope under heavy rain.

Research indicators	Principal component		communality
	1	2	
Root biomass	0.447	0.895	0.998
Total root length	0.863	0.505	0.997
Total root volume	0.893	0.449	0.995
Total root surface area	0.865	0.501	0.992
Characteristic value	3.669	0.329	
Variance %	91.725	8.232	
Cumulative variance%	91.725	99.957	

**Table 5:** Principal component rotation factor load and public factor variance of shrub root morphological traits at 60 slope under heavy rain.

Research indicators	Principal component		communality
	1	2	
Root biomass	0.109	0.986	0.983
Total root length	0.974	0.205	0.990
Total root volume	0.962	0.173	0.955
Total root surface area	0.987	0.162	0.997
Characteristic value	3.070	0.858	
Variance %	76.742	21.461	
Cumulative variance%	76.742	98.203	

## 5. Discussion

Different shrub roots play different reinforcement roles in improving the stability of ecological slope [19]. Under the same conditions, if the shrub can improve the soil scour resistance, then the slope damage rate will be reduced [20]. In this study, shrubs, better adapted to improve the structural stability of the slope, can be obtained by comparing the relationship between root morphological characteristics and soil anti-scouring resistance of AFL, SOL and LBT under different rainfalls and different slopes.

### 5.1. Comparted soil anti-scouring resistance index in different shrubs

In this study, under the two conditions of light rain and heavy rain, the soil anti-scouring resistance index of SAO was significantly higher than that of AFL and LBT with slopes of both 30° and 60°. This suggested that plant species played an important role in explaining soil anti-scouring resistance. Previous studies found that there was a linear positive proportional relationship between the increase of the soil anti-scouring resistance and root biomass [21]. At the same time, roots can directly change the soil pore structure, helping to form a large connected network, promote free water to quickly enter and cross the soil profile, reduce the water pressure in the soil, and improve the soil anti-scouring resistance [22, 23]. Since the individual root biomass of the SAO was large, this resulted in greater root-soil contact area than the other two shrubs [24]. Therefore, the soil anti-scouring resistance index of SAO was larger than that of the other two shrubs studied.

In this study, the soil anti-scouring resistance index decreased significantly with increasing slope when the plant species were the same. Plant type, slope, and its interaction had significant effects on all soil anti-scouring resistance index for all slopes. This suggested that the slope can significantly affect the soil anti-scouring resistance. This was consistent with previous studies [25, 26]. Ziadat and Taimeh (2013) found that, the soil anti-scouring resistance of non-cultivated soil were mainly affected by slope [27]. Previous studies found that the relationship between scour load and slope were significant [28, 29].

In this study, the soil anti-scouring resistance index under heavy rain was significantly lower than that under light rain. This indicated that the rainfall was an important factor affecting soil anti-scouring resistance index. This was consistent with previous studies. Previous studies found that with the rainfall increased, the soil anti-scouring resistance index gradually decreased [30]. Ziadat and Taimeh (2013) also found significant effects of rainfall on soil anti-scouring resistance [27]. Zeng found that rainfall was inversely proportional to soil anti-scouring resistance [31]. The reason for this result might be that rainfall and duration were important factors in controlling soil erosion rates [32–34]. In a certain slope range, with the increase of rainfall, the slope runoff rate increased, so that the erosion force of the slope runoff increased, which leads to the mechanical damage capacity and soil handling capacity of the ground runoff produced by rainfall, so the soil erosion amount increased [35].

### 5.2. Relationship between soil anti-scouring resistance index and root biomass

In this study, under the two conditions of light rain and heavy rain, there was a positive correlation between soil anti-scouring resistance index and root biomass with slopes of both 30° and 60°. This was consistent with previous studies [36]. With the increase of plant root biomass, the soil anti-scouring resistance of slope increased [37]. The reason for this result was that when the shrub root biomass was small, it was difficult to form a larger network structure, so the root was not easy to enhance the impact anti-scouring resistance effect of the root-soil complex [38]. The biomass can increase the soil fixation capacity, and the fine roots existing in the soil layer can aggregate the soil, increase the soil anti-scouring resistance, and reduce the soil scour caused by surface runoff [39].

In this study, under the same biomass conditions, AFL had the largest soil anti-scouring resistance index, followed by SAO and finally LBT. This might be due to the differences in the root distribution and morphology of the shrubs. SAO had no obvious taproots, and more fine roots [18]. LBT had obvious taproots and fewer lateral roots, the root mean diameter was smaller than AFL, and fewer fine roots than AFL. The taproot of AFL had a large number of lateral roots, and the taproot and lateral root interlaced, which can very effectively wrap the soil, forming the soil form with strong anti-scouring resistance, thus playing a better soil consolidation enhancement effect. However, increasing planting density can enhance soil reinforcement, but it can also increase soil infiltration under rainfall, which is not beneficial for soil stability [63]. When the root content is small, the porosity of the soil layer can be increased by increasing the plant root density, thus increasing the permeability coefficient of the soil. However, when the plant root density is increased to a certain value, the permeability of the soil layer will also be reduced to a certain extent because the plant root will gradually cover the previously dispersed and compacted soil layer. The formation of plant roots generally increases the content of water stable aggregates, increases the porosity of the soil layer, and thus increases the soil permeability coefficient. Therefore, the enhancement of plant roots increases the permeability of soil, thus improving the anti-scourability of soil, and achieving the effect of restraining surface runoff.

### 5.3. Relationship between soil anti-scouring resistance index and root morphological traits

TRL, TRV, and TRSA area reflected the distribution characteristics of plant roots in soil [40]. It was closely related to the soil anti-scouring resistance [41, 42]. Therefore, by studying the morphological traits of plant roots, we can evaluate the ability of the roots for soil consolidation [43]. In this study, under the two conditions of light rain and heavy rain, there was a positive correlation between soil anti-scouring resistance index and total root length, total root volume, and total root surface area with slopes of both 30° and 60°. This indicated that the root morphology traits influence the soil anti-scouring resistance index. This was consistent with previous studies [44]. Previous studies found that the morphology of the plant roots was related to the soil anti-scouring resistance [44]. Some scholars found that TRL, TRV, and TRSA were positively correlated with the soil anti-scouring resistance index [18]. The reason for this result might be that the root length, volume, and surface area of the root system directly affected the size of the root-soil contact area and the friction between the root and soil [45, 46]. It was also reported that with the increase of TRL, TRV, and TRSA, root secretion increased, soil cohesion increased, and soil anti-scouring ability to resist ground runoff increased [47].

This study found that under the same root morphological traits, AFL had the largest soil anti-scouring resistance index followed by SAO and finally LBT. The reason for this result might be different root architectural traits in different plants [48]. Previous studies found that under the condition of similar root morphology traits, root architectural traits had a great impact on the enhancement of soil consolidation [49]. The root branch density can especially increase the soil anti-scouring resistance of the root-soil complex [50, 51]. SAO had a relatively high branching density of roots in high-order roots, but a relatively low branching density of roots in low-order roots [52]. AFL had a relatively high branching density of roots in both high and low-order roots with a relatively large branching angle of roots [52]. In conclusion, the soil anti-scouring resistance index of AFL was largest with the same TRL, TRV, and TRSA.

### 5.4. Main factors affecting the soil anti-scouring resistance index

In this study, under the two conditions of light rain and heavy rain, root morphological traits played a key role on soil anti-scouring resistance with slopes of both 30° and 60°. This was in agreement with previous studies. Soil anti-scouring resistance was the ability of the soil to resist the mechanical damage of ground runoff [53], which was closely related to plant root morphological traits [50, 51]. Previous studies indicated that the reinforcement of soil by plant roots mainly depended on several variables such as root system morphology and mechanical architectures [54, 55]. Root morphological traits can significantly change the distribution of stresses and plastic strains within the soil medium as well as affect the resistance to scouring [56]. Different root morphological traits had effects on the structure of the root-soil complex, causing the difference in the soil anti-scouring resistance [57]. Root morphological traits directly reflected the ability of roots to absorb nutrients and water, which clearly affected soil porosity and increased the resistance to erosion [58]. In quantifications of the effects of roots on runoff and soil loss, the distribution density of root systems often acted as a major indicator, including TRL, TRV, and TRSA [59–63].

## 6. Conclusions

In this study, we selected three shrubs with similar life types under two slopes and two rainfall conditions to reveal the relationship between soil anti-scouring resistance and root biomass and root morphological traits. Through this study, the following conclusions are observed from the study.

- Under two slopes and two rainfalls conditions, the soil anti-scouring resistance index of three shrubs varied significantly. For individual plants, SAO had the highest soil anti-scouring resistance index. These results indicated that anti-scouring resistance of SAO was best in the case of individual plants.
- Under two slopes and two rainfalls conditions, AFL had the highest soil anti-scouring resistance index when root biomass and root morphological traits were identical. These results indicated that AFL exhibited the best anti-scouring resistance when the root biomass and root morphological traits were the same. Therefore, in the engineering practice, the soil anti-scouring resistance can be increased by selecting the planting AFL.
- Under two slopes and two rainfalls conditions, RB and root morphological traits of three shrubs were significantly correlated with the soil anti-scouring resistance index. Therefore, we can use RB, TRL, TRV, and TRSA to reveal the action mechanism of shrub root on soil anti-scouring resistance.
- Under two slopes and two rainfalls conditions, according to the composition characteristic values and the contribution rate, the three-shrub root morphological factors have high contribution rate. Therefore, the root morphological factor was the key factor affecting the soil anti-scouring resistance. These results indicated that TRL, TRV, and TRSA played an important role in the enhancement effect of soil anti-scouring resistance.

## Supporting information

S1 File (DOCX)

S1 Data (XLSX)

S2 Data (XLSX)

S3 Data (XLSX)

S4 Data (XLSX)
