# Supplementary material for: Root biomass and root morphological traits of three shrub species: Implications for the soil anti-scouring resistance of the ecological slope
Source: PLoS One. 2023 Nov 16;18(11):e0288848. doi: 10.1371/journal.pone.0288848 (PMC10653482; doi:10.1371/journal.pone.0288848)
Supplement: S1 File — (DOCX) [file pone.0288848.s001.docx]

**Root Biomass and Root Morphological Traits of Three Shrub Species: Implications for the Soil Anti-scouring Resistance of the Ecological Slope**

Mingxin Zhou^1,3,4^, Guoyong Yan^2,^* , Yibo Li^3,5^, Di Chen^1^, Chao Yan^1^, Nan Wang^4^, Chao Jia^4^, Qinggui Wang^2^, Yajuan Xing^2^

^1^ Heilongjiang Academy of Forestry, Harbin, China.

^2^School of Life Sciences, Qufu Normal University, Qufu, China.

^3^ Northeast Forestry University, Harbin, China.

^4^Heilongjiang Institute of Construction Technology, Harbin, China.

^5^ Heilongjiang Polytechnic, Harbin, China.

*Corresponding authors: Guoyong, Yan, guoyongyan1991@163.com


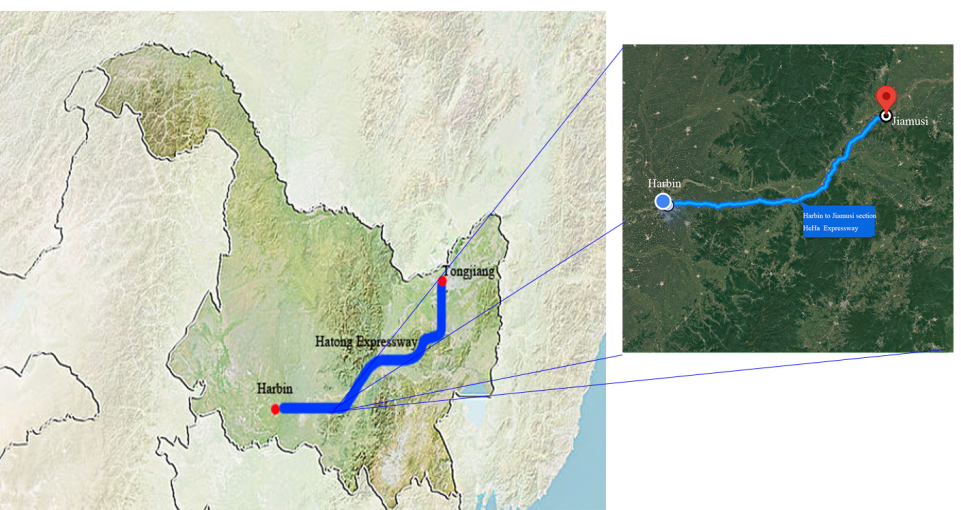


**Figure S1.** The map of the study area, which is in the Hatong Expressway form Harbin to Jiamusi

**
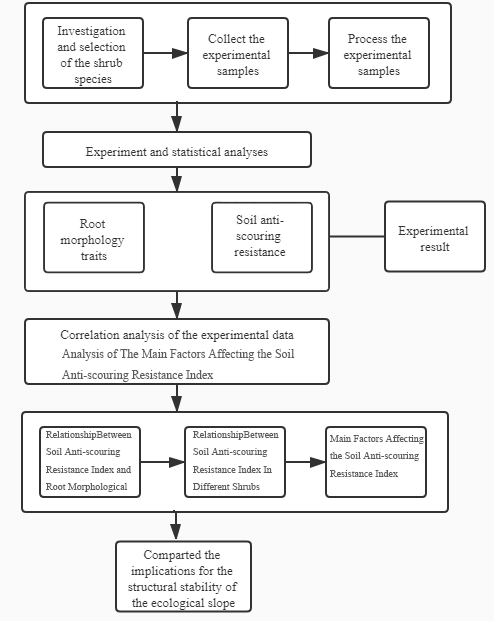
**

**Figure S2.** The flowchart of the methodology


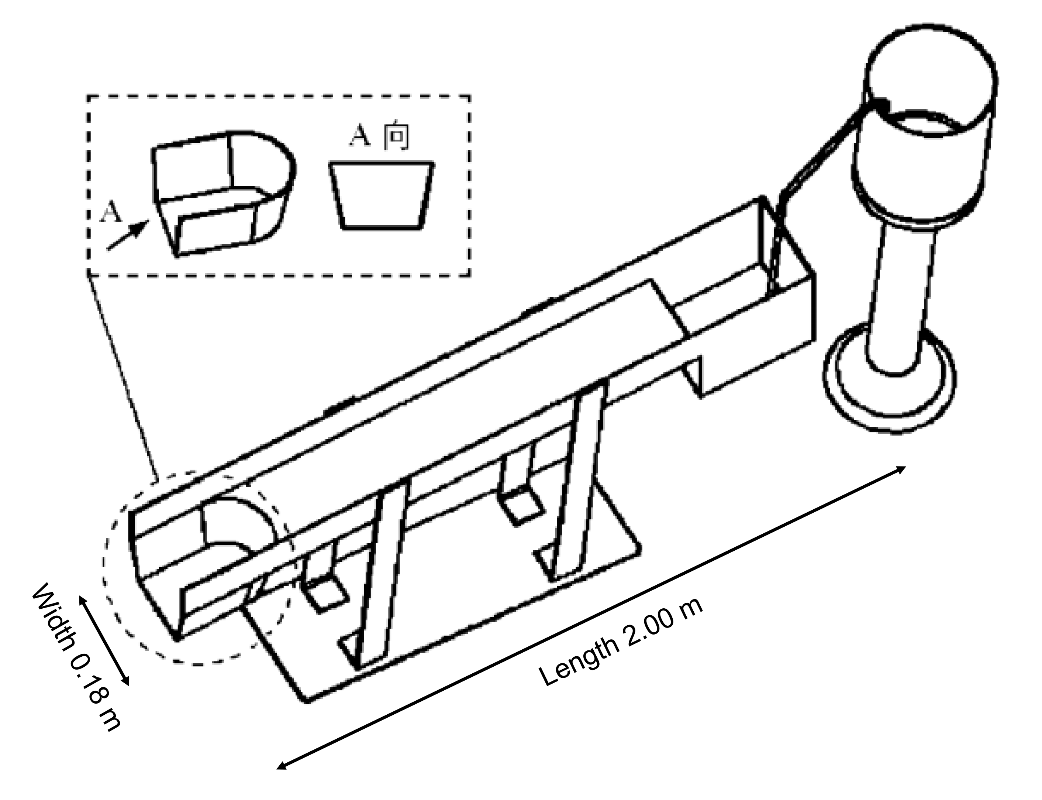


**Figure S3**. The experiment device based on the original soil wash tank. A, a 20cm × 18cm × 10cm sampler.


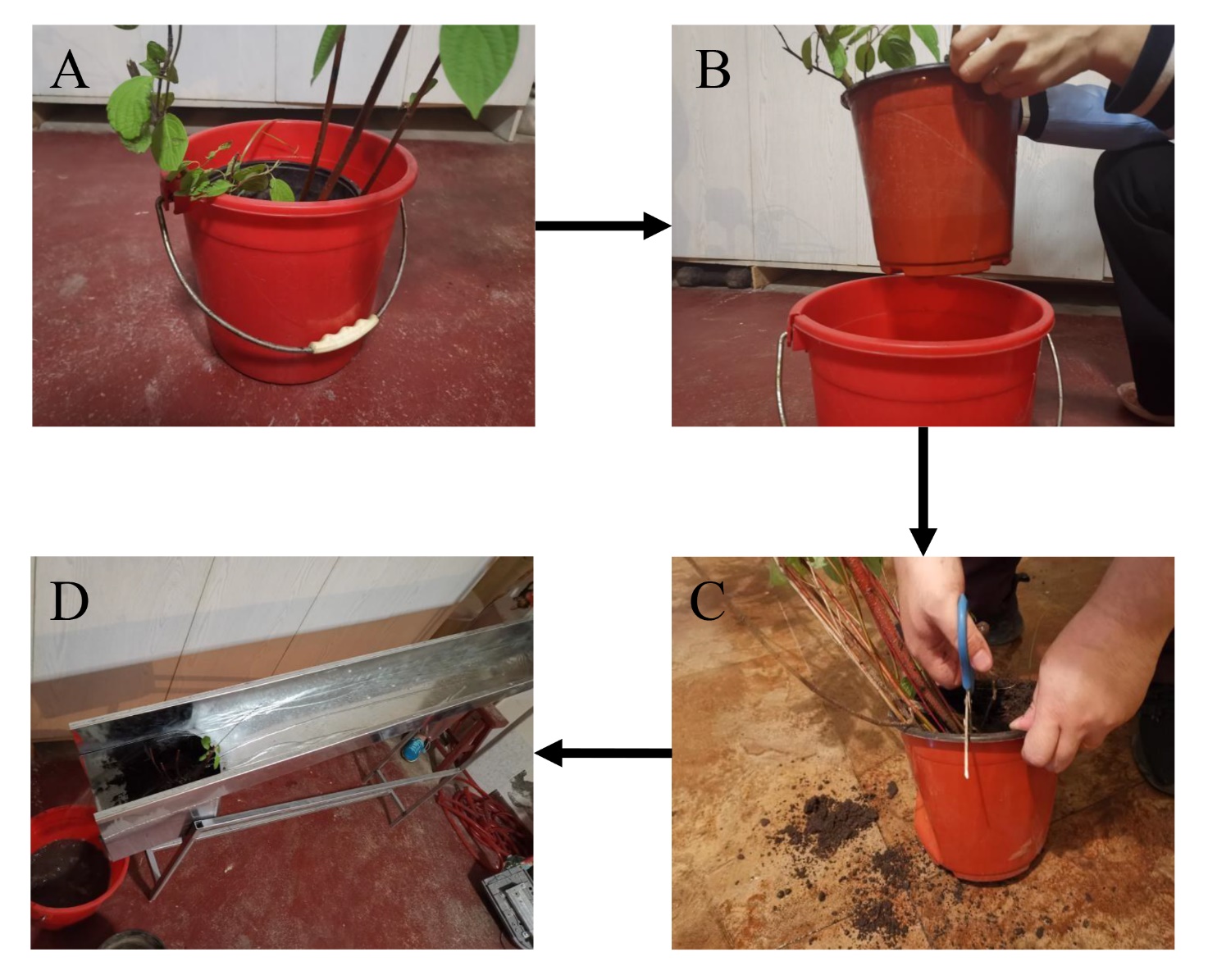


Figure S4. The specific operation steps of soil anti-scouring resistance index determination. (A) Before the scouring test, put the potted plants on the water surface and let the soil absorb water to saturation; (B) After taking out the potted plants, first stand and remove the gravity water until no obvious water drops flow out to obtain the same soil moisture content; (C) Cut the plastic flowerpot, cut off the aboveground part of the plant, use the soil sampler to take out the soil block with complete plant roots, and put the soil block with complete plant roots together with the soil sampler into the washing tank for washing; (D) then, we collect and record the amount of soil scoured in unit time, dry at 105 ℃, and weigh, and record it as ΔW (mass of soil washed away during scouring).

Table S1 The soil permeability coefficient of roots under different root content (20℃)

| Root biomass (%) | soil permeability coefficient（10^-4^cm/s） | Permeability coefficient increment（10^-4^cm/s） | Permeability coefficient increase（%） |
| --- | --- | --- | --- |
| 0 | 0.832 | 0 | 0 |
| 0.1 | 1.088 | 0.256 | 30.8 |
| 0.2 | 1.765 | 0.933 | 112.1 |
| 0.25 | 2.123 | 1.291 | 155.2 |
| 0.3 | 1.675 | 0.843 | 101.3 |
| 0.35 | 1.487 | 0.655 | 78.7 |
| 0.4 | 1.201 | 0.369 | 44.3 |
